# The Mechanical Behaviors of Polyethylene/Silver Nanoparticle Composites: an Insight from Molecular Dynamics study

**DOI:** 10.1038/s41598-020-64566-4

**Published:** 2020-05-05

**Authors:** Chia-Hao Su, Hui-Lung Chen, Shin-Pon Ju, Hsing-Yin Chen, Che-Wei Shih, Cheng-Tang Pan, Tai-Ding You

**Affiliations:** 1 Institute for Translational Research in Biomedicine, Kaohsiung Chang Gung Memorial Hospital, Kaohsiung, 833 Taiwan; 2 Department of Chemistry and Institute of Applied Chemistry, Chinese Culture University, Taipei, 111 Taiwan; 3 Department of Mechanical and Electro-Mechanical Engineering, National Sun Yat-sen University, Kaohsiung, 80424 Taiwan; 4 Department of Medicinal and Applied Chemistry, Kaohsiung Medical University, Kaohsiung, 807 Taiwan

**Keywords:** Polymers, Nanoparticles

## Abstract

This research uses molecular dynamics simulation (MD) to study the mechanical properties of pristine polyethylene (PE) and its composites which include silver nanoparticles (PE/AgNPs) at two AgNP weight fractions of 1.05 wt% and 3.10 wt%. The stress-strain distribution of the tensile process shows that the embedded AgNPs can significantly improve the Young’s modulus and tensile strength of the pristine PE, due to improvements in the local density and strength of the PE near the AgNP surface in the range of 12 Å. Regarding the effect of temperature on the mechanical properties of pristine PE and PE/AgNP composites, the Young’s modulus and the strength of the pristine PE and PE/AgNP composites decreased significantly at 350 K and 450 K, respectively, consistent with predicted melting temperature of pristine PE, which lies at around 360 K. At such temperatures as these, PE material has stronger ductility and a higher mobility of AgNPs in the PE matrix than those at 300 K. With the increase of tensile strain, AgNPs tend to be close, and the fracture of PE leads to a similarity between both the Young’s modulus and ultimate strength found for the pristine PE and those found for the PE/AgNP composites at 350 K and 450 K, respectively.

## Introduction

The development of polymer-based composites by the addition of different fillers has proved to be a promising approach to alter the materials properties of polymers, and this manufacturing method makes it possible for new composites to be used in industrial applications^1,2^. For example, properties found in pristine polymers like mechanical strength^3^, elasticity^4,5^, plasticity^6,7^, electrical conductivity^8,9^, and thermal conductivity^10,11^, can be significantly enhanced when the polymer matrixes undergo mixing with filler material that has been well-dispersed.

Among all kinds of thermoplastic polymers, polyethylene (PE) has found wide use due to its excellent mechanical properties. Among these are specific mechanical properties like a high ductility of 6MN/m^2^, such that polyethylene can be stretched to 103% of its length before cracking, unlike some metals, which can withstand only 100.71%. Another mechanical property of PE, impact strength, is more than 90 N/cm^2^ ^12^, the highest among thermoplastic polymers. Polyethylene’s heat resistance is excellent, with thermal stability of around 70 to 110 °C^13^, and its melting point is typically 105 to 115 °C^14^. Consequently, many potential fillers such as a poly(HEMA) matrix^15^, carbon fiber^16^, and clay (montmorillonite)^17^, have been mixed with pristine PE to achieve specific improvements. These composites have been used extensively in the automotive industry in reinforced glass and electrical insulation, and in biomedical applications such as artificial joints^18^. Many experimental methods have been carried out to study the properties of PE composites. For example, Nawang showed that the mechanical properties such as yield strength and tensile strength of starch or linear low density polyethylene (LLDPE) composites decreased with an increase in filler content, with the optimum filler content being 15%^19^. Juhasz found that, when glass-ceramic particle size was increased to 4.4 and 6.7 μm, the Young’s modulus, yield and bending strengths were slightly reduced. A transition in fracture behavior from ductile to brittle behavior was observed in samples containing between 30 and 40 vol% filler^20^. Rusu showed that, for HDPE/zinc powder composites (zinc = 0 to 20% by volume), an increase in zinc powder content in the composites induced an increase in brittleness^21^.

In addition to the conventional filler materials above, it has been reported that nanostructure filler materials such as nanowires^22^, nanotubes (NTs)^23,24^, nanorods, and nanoparticles^25–27^ can also enhance some polymer properties more significantly than bulk fillers. For example, Tang examined a multi-wall carbon nanotube (MWNT)/HDPE composite with 0, 1, 3 and 5% nanotube content by weight, with results showing increases in the stiffness and peak load with increasing MWNT content^24^. Zhang investigated nano-SiO2 particle/HDPE composites, with results showing that when nano-silica particles comprised 0.75 vol%, tensile stiffness, tensile strength and impact strength of the composites increased^28^. Nurazreena studied the mechanical properties of HDPE/metal nanoparticle composites (aluminum, copper, and iron nanoparticles, with content varying from 10% to 55% by volume) by tensile testing. The results show that the Young’s modulus of HDPE can be increased by adding metal powder into HDPE, while the tensile strength of HDPE filled with Al and Fe reached the maximum value at 10%, while the maximum value for Cu-HDPE is at 20%^29^. Beside the studies about the effect of nanofiller on the mechanical property of composites, Ansar conducted a series of MD simulations to find the methods to enhance the mechanical properties of several nanofillers^30–34^. These modified nanofillers were further used to improve the mechanical properties of composites. For example, in Ajori’s study^35^, their MD simulation results showed the NW-encapsulated CNT, one of the modified nanofiller of Ansar’s studies, considerably increases the buckling force and strain of the metallic glass composites.

Previous research has confirmed that Ag nanoparticles (AgNPs) are superior to many other metal nanostructure particles due to their unique electrical, chemical, mechanical, and optical properties^36–38^. It has strong antimicrobial and specific inhibiting effects^39^, which support numerous research and practical applications^40^. Therefore, PE matrix seems likely to demonstrate improved mechanical properties by adding Ag nanoparticle filler^41^. For example, Valdes investigated PE-silver nanoparticle composites containing either 1% or 0.6% silver nanoparticle content, and results indicated that mechanical properties (tensile strength, elongation at break) were improved at 1%^42^. Aalaie studied polyethylene–silver nanoparticle compounds with a range of 0–2% silver nanoparticle content, and their results show that reinforcements with as little as 1 wt% of nanosilver provided effective antibacterial performance while generally improving the compound’s mechanical properties^43^.

These experimental results show that the mechanical properties of pristine PE can be improved by adding AgNP as a composite PE/AgNP. However, it is difficult to obtain a mechanism of interaction between PE and AgNP which causes mechanical property enhancement by using an experimental method. Therefore, certain computer simulation approaches can be effective alternative methods to successfully demonstrate the mechanism of interaction between PE and AgNP at the atomic scale. Among these, molecular dynamics (MD) simulations are a powerful investigative method for studying the interaction between atoms on the basis of exact interaction potential. Yang, using MD simulations, investigated the physical behavior of the defects affecting Thrower-Stone-Wales (TSW) composites as well as the Young’s modulus of CNT/polypropylene (PP) composites and the transverse and longitudinal shear moduli of the composites that result from the increased stronger interfacial adhesion between defect CNTs and the matrix^44^. Adnan used a polyethylene (PE) matrix with three different diameters (0.7, 1.2 and 1.7 nm) of nano-scale buckyballs to study the effect of nanoparticle size on the elastic properties of polymer nanocomposites by MD simulations. Their results show that the elastic properties of the nanocomposites show clear improvement with the decrease of the size of the buckyball^45^. Frankland studied two NT geometries, those of long continuous fibers and short discontinuous fibers, and results indicated that the long-nanotube composite shows an increase in the stiffness relative to the polymer^46^.

In Aalaie’s study^43^, the experimental tensile strength increases very slightly with the AgNP fraction ranging from 0 to 1.5%, so the mechanical property improvement content could be the margin of error. They also stated the mechanical improvement of polymer by adding nanofiller is more significant when the fraction of nanofiller exceeds 2 wt%. To understand the interaction between PE and AgNP, the atomic scale simulation method is necessary. However, as far as we know, the physicochemical properties of the polyethylene /silver (PE/Ag) nanoparticle composite have not been explored by simulation technology. In this paper, we employ LAMMPS (large-scale atomic/molecular massively parallel simulator) to calculate the model of PE/Ag nanoparticle composites for a comparison with pristine PE. The most stable molecular configuration with global energy minimization and mechanical properties of nanocomposites are evaluated. These significant results could find application in polymer/nanoparticle systems currently under development. The same simulation process has been successfully used to investigate mechanical properties of polymethylmethacrylate/silver nanoparticle composites in our previous study^47^. We believe this molecular simulation process can clearly explore the local structural deformation at the atomic scale during the tensile loading.

## Materials and Simulation Methods

**Figure 1:**
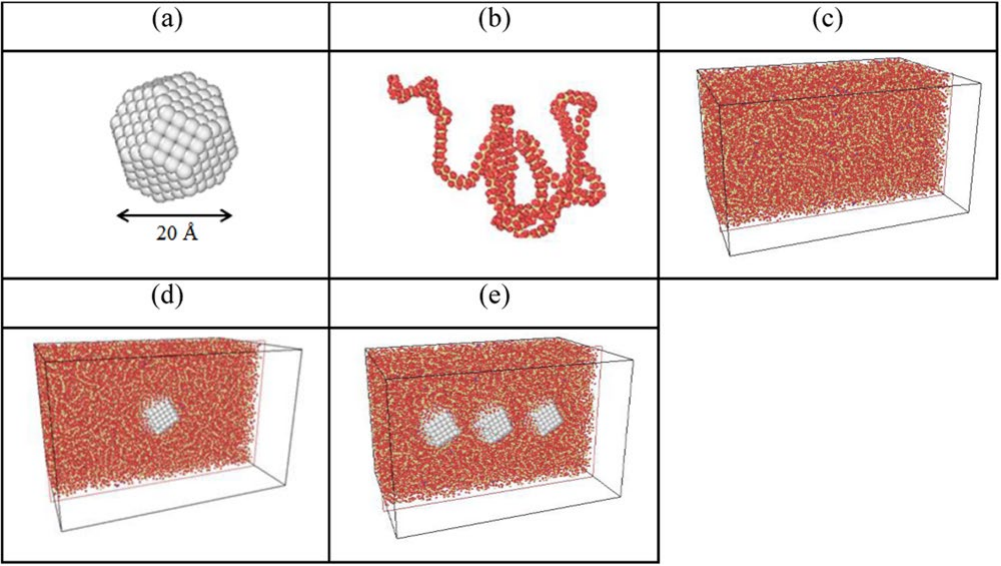
Schematic diagrams of (**a**) Ag nanoparticle, (**b**) a PE chain, (**c**) PE model, as well as (**d**) PE/AgNP (1.05%) and (**e**) (3.10%) models.

To explore mechanical properties of pristine PE and PE/AgNP composites, molecular dynamics simulations were performed using LAMMPS^48^. Figure 1(a–e) show the schematic diagrams of one AgNP, a PE chain, a pristine PE model, and PE/AgNP composites with one and three AgNPs, respectively. The PCFF (polymer consistent force field) force field^49^ is used to in order to describe interactions between PE atoms, as PCFF is used for parameterization and verification of PE, including such characteristics as bond stretching and angle bending, as well as dihedral and improper interactions and coupling between them. Electrostatic and van der Waals interactions are the non-bonded terms, with the latter represented here by the Lennard-Jones (LJ) 9–6 potential. In addition, PPPM (particle-particle-particle-mesh) summation is used to evaluate long-range electrostatic interactions.

The interaction between Ag atoms in AgNP was simulated by using the many-body tight-binding potential. This model sums the band energy and is characterized by the second moment of the d-band state density a pair of potential energy of the Born-Mayer type, i.e.,

1 $$Ei=-\,{\left\{\sum _{j}{\xi }^{2}\exp \left[-2q\left(\frac{rij}{r0}-1\right)\right]\right\}}^{1/2}+\sum _{j}A\exp \left[-p\left(\frac{rij}{r0}-1\right)\right]$$

where *ξ* is the effective hopping integral, *r*_*ij*_ is the distance between atoms *i* and *j*, and *r*_0_ is the first-neighbor distance. The parameters of the Ag-Ag interactions used in this present study are listed in Table 1 ^50^.

**Table 1:** The tight-binding potential parameters of Ag.

	*A* (eV)	*ξ* (eV)	*p*	*q*	*r*_0_ *(Å)*
Ag	0.103	1.178	10.928	3.139	2.89

Table 2 shows the lattice constant, binding energy, elastic constants (C11, C12, and C44), and bulk modulus obtained by the TB potential and experiment as well as the error between the corresponding data. It can be seen the predicted and experimental data are almost identical, indicating the TB potential can accurately describe the structural and mechanical properties of Ag material. In Lobato’s study^51^, the TB potential was used to investigate the local structural transformation during the cooling processes with different cooling rates. In Ngandjong’s study^52^, the TB potential was used to describe the structural behaviors of AgNP on the silica surfaces with different surface roughness. In Sauceda’s study^53^, the TB potential was used to obtain the vibrational density of states of AgNP. The vibrational density of states of icosahedral AgNP predicted by the TB potential closely matches that from the experimental measurement. From the aforementioned studies, it is proposed that the MD simulation using the TB potential can accurately reflect the structural and mechanical properties of AgNP in the current study.

**Table 2:** The material properties of Ag obtained by tight-binding potential calculation^50^ and experimental measurement^63^.

	TB potential	Experiment	Error (%)
Lattice constant (Å)	4.085	4.09	0.12
Binding energy (eV/atom)	2.96	2.95	0.34
Elastic constant C11(GPa)	132	131	0.76
Elastic constant C12(GPa)	97	97	0.00
Elastic constant C44(GPa)	51	51	0.00
Bulk modulus (GPa)	108	108	0.00

To accurately model the interaction between AgNP and PE, the optimized PCFF force field, as developed in research by Heinz *et al*., was used^54^. The optimized PCFF force field accurately simulates the interfaces between metal and hybrid materials and inorganic, organic, and biological compounds. It also more clearly illustrates the various roles in the mixed (metallic/nonmetallic) system.

For the pristine PE system, the simulation system was constructed using the PE chain containing 150 monomers, for a total of 108 PE chains. Because of the computational limitation on the molecular dynamics simulation, most polymer models are much shorter than the real ones used in the related experiment. The alternative way is to use a polymer model, which can predict the identical material properties as the longer polymer model is used. In the current study, mechanistic properties of PE/AgNP composites are very similar to those by the PE model longer than 150 monomers. The procedure for obtaining the equilibrium configuration is described as follows: first, the structure is optimized using the conjugate gradient method, with energy convergence condition set at 10^−6^ kcal/mol, and maximum iterations of 10^5^. After this optimization, the system reached equilibrium at a temperature of 1000 K for a time period of 100 ps at 1 atm. Next, the system was quenched from 1000 K to 300 K at a cooling rate of 3.5 K/ps at 1 atm pressure using an isobaric-isothermal ensemble (NPT). Finally, MD simulation of the other 50 ps was carried out at 300 K and 1 atm by NPT integration in order to achieve the equilibrium condition. Within the final 50 ps, both the temperature and pressure fluctuate at constant values, indicating the system has completely relaxed. The density of the pristine PE system obtained by simulation of the annealing was about 0.8 g/cm^3^, representing a decrease of about 12% from the corresponding experimental value of about 0.91 ~ 0.94 g/cm^3^^12^.

For PE/AgNP composites, the AgNP with a radius of about 10 Å was composed of 405 Ag atoms. In order to investigate the effect of Ag atomic fraction on the PE/AgNP mechanical properties, PE matrixes containing either one or three AgNPs were considered, with their corresponding weight fractions being 1.05% and 3.10%, respectively. These composites with different weight fractions are here termed AgNP (1.05%) and AgNP (3.10%) and are used to describe our simulation results. In order to insert AgNP into the PE matrix, a hollow spherical space was formed in the inner region of the PE matrix by extruding a virtual repulsive sphere with a radius from 0.1 to 12 Å. The radius of the sphere itself is 12 Å. The increment by which the radius of the virtual repulsive spherical surface was increased was about 0.01 Å, and geometrical optimization was carried out before the application of the subsequent increment. After the AgNP was located within the virtual sphere, the system underwent annealing from an initial temperature of 1000 K, down to 300 K with a cooling rate of 3.5 K/ps. The PE/AgNP interface was then relaxed by the NPT system at pressure of 1 atm. The Nosé-Hoover thermostat and barostat^55^ were utilized throughout the MD simulation, with Table 3 presenting detailed information of the system sizes at a strain of 0 for the three simulation models, including PE chain number, the Ag atom number, and the total atom number.

**Table 3:** Detailed simulation information for pristine PE, PE/AgNP (1.05%), and PE/AgNP (3.10%).

	box dimensions [Å^3^]	Number of PE chains	Number of Ag atoms	Total atom numbers
PE	137.7 × 93.2 × 75.9	108	0	97,416
PE/AgNP (1.05%)	134.4 × 87.8 × 83.2	108	405	97,821
PE/AgNP (3.10%)	135.5 × 87.6 × 83.3	108	1215	98,631

During the tensile simulation, the increment in the x dimension is 0.25% of x length at strain of 0. After each tensile increment, the system was relaxed by using 20 ps MD simulation. At the last 5 ps of the relaxation process, the tensile stress was calculated by Eq. (2) ^56^:

2 $${\sigma }_{mn}=\frac{1}{{V}_{s}}\sum _{i}\left[{m}_{i}{V}_{i}^{m}{V}_{i}^{m}-\frac{1}{2}\sum _{j}\frac{\partial \varnothing ({r}_{ij})}{\partial {r}_{ij}}\frac{{r}_{ij}^{m}{r}_{ij}^{n}}{{r}_{ij}}\right]$$

where ${m}_{i}$ is the mass of atom *i*, ${V}_{s}$ is the system volume, *r*_*ij*_ is the distance between atoms *i* and *j*; and ${r}_{ij}^{m}$ and ${r}_{ij}^{n}$ are two components representing the vector from atoms *i* to *j*.

## Results and Discussion

**Figure 2:**
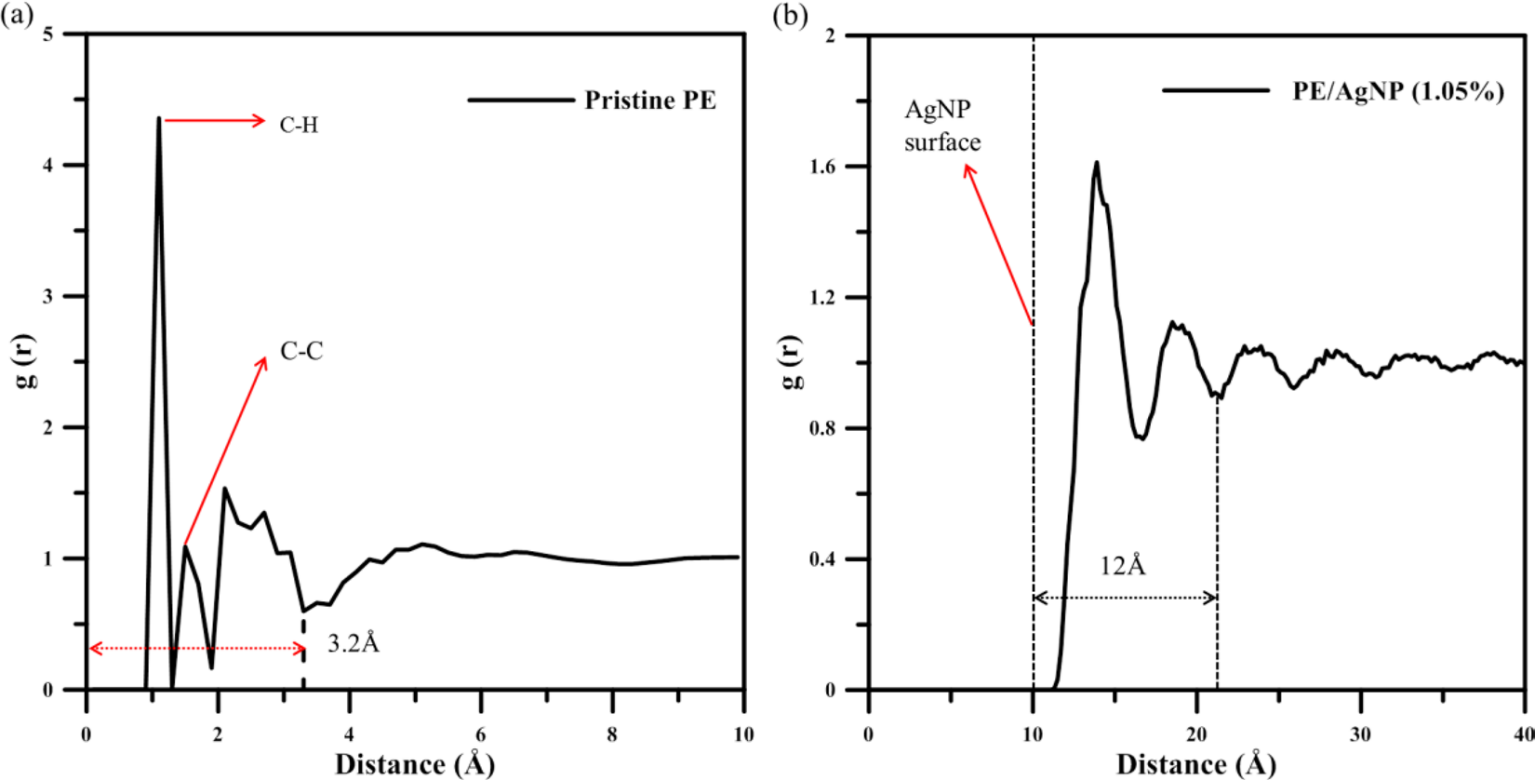
Radial distribution functions (RDFs) for (**a**) pristine PE and (**b**) PE/AgNP (1.05%). It should be noted that the origin of PE/AgNP (1.05%) RDF is the mass center of AgNP.

Figure 2 presents the radial distribution functions (RDFs) of the PE/AgNP systems for all pristine PE atom pairs as well as PE/AgNP pair types. Note that the RDF origin in this PE/AgNP system is located at the center of mass of AgNP. In Fig. 2(a), the RDF curve shows two different narrow peaks appearing at 1.1 Å and 1.7 Å, which represent the bonded C-H pairs and C-C pairs, respectively. The wider peak, along the range 1.7 to 3.2 Å, is the first peak in the non-bonding RDF profile, which indicates that PE has an influence distance of 3.2 Å for the non-bonding interaction around one atom. For the RDF profile of PE/AgNP composite shown in Fig. 2(b), the origin is located at the mass center of AgNP and the AgNP surface is indicated by the dashed line at 10 Å. One can see the first two RDF peaks at 14 Å and at 18 Å close to the AgNP surface are more distinct, compared to peaks at the distance longer than that of the second RDF peak. Consequently, it was proposed that the AgNP interaction on PE is more significant within the distance of 12 Å from the AgNP surface as indicated by the distance between two arrows. Because the RDF value of the first peak is the most prominent, it indicates the PE chain atoms located within 4 Å from AgNP surface have strong interaction strengths coordinating to AgNP, as well as having a high local density.

**Figure 3:**
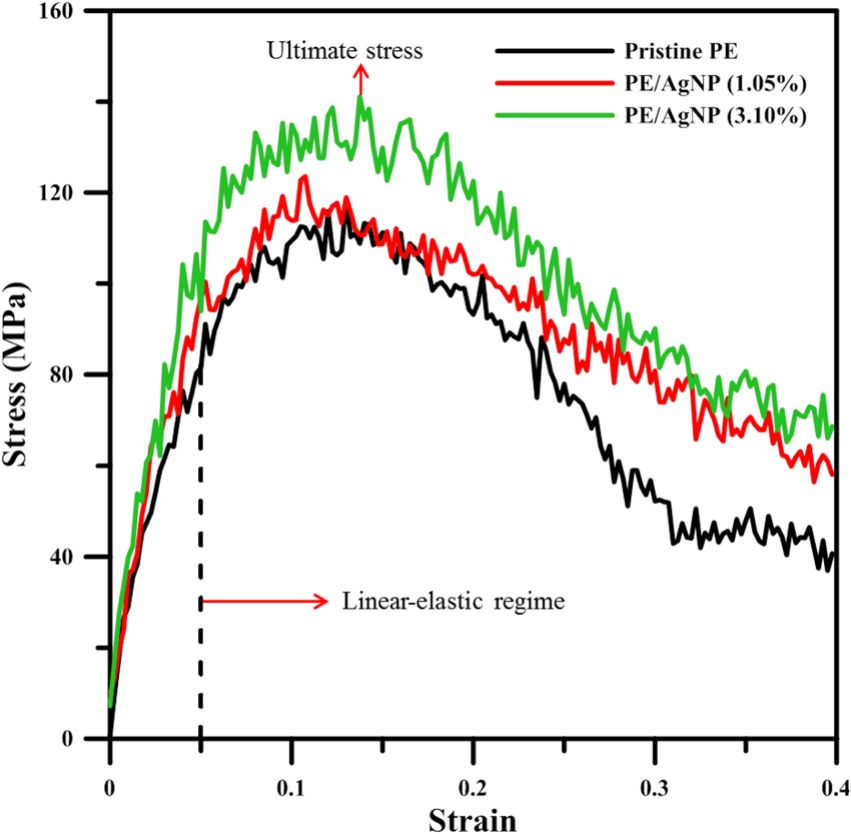
Stress-strain curves of pristine PE, PE/AgNP (1.05%) and PE/AgNP (3.10%).

Stress-strain curves, presented in Fig. 3, describe Ag atoms under tensile loading at a temperature of 300 K for pristine PE and AgNP (1.05%)/AgNP (3.10%). From 0 to about 0.05, the stress exhibits linear proportionality to the strain, then increases parabolically with an increase in strain up to the ultimate stress point. After strain exceeds this ultimate stress point, stress exhibits a gradual decrease in all three cases. The ultimate stresses of both PE/AgNP composites reach a higher level than that of pristine PE, with that of AgNP (3.10%) (at the highest Ag wt%) being the largest.

The mechanical properties, derived from these stress-strain profiles, are listed in Table 4, with the Young’s modulus having been obtained from the slope of a linear fitted line using data for strains smaller than 0.02. The Young’s moduli of pristine PE, PE/AgNP (1.05%), and PE/AgNP (3.10%) are about 1495.16 MPa, 1689.26 MPa, and 1830.76 MPa, respectively. The increase in Young’s modulus of PE/AgNP (1.05%) and PE/AgNP (3.10%) are about 11% and 18% of that of pristine PE. Compared to mechanical properties of PE/CNT composites, in Frankland’s^46^ and Xie’s^57^ studies, they stated the mechanical properties of PE are almost unchanged after the CNTs was randomly blended with PE. Since the CNT is a nanofiller with a high aspect ratio, Haghighatpanah’s simulation results show the Young’s modulus of PE/CNT along the longitudinal direction of CNT has a 380% increase and that along transverse direction of CNT display a 52% increase^58^, compared to that of pristine PE. To obtain the toughness of PE, PE/AgNP (1.05%) and PE/AgNP (3.10%), the stresses of stress-strain curves were integrated from the strain of 0 to that of ultimate stress. The calculated toughness values of PE, PE/AgNP (1.05%) and PE/AgNP (3.10%) are about 30.2, 35.05 and 39.96 MJ/m^3^. It can be seen the toughness values of PE/AgNP (1.05%) and PE/AgNP (3.10%) are improved by 16.05% and 32.3% as compared to that of pristine PE.

**Table 4:** Young’s modulus and ultimate stress for pristine PE and PE/AgNP at different Ag weight fractions.

**AgNP weight fraction (%)**	**0**	**1.05**	**3.10**
Young’s modulus (MPa)	1495.16	1689.26	1830.76
Ultimate stress (MPa)	118.55	123.56	141.09

In addition, in terms of ultimate stress or tensile strength, Table 4 illustrates that the tensile strength is enhanced from 118.55 Mpa for pristine PE to 123.56 MPa or 141.09 MPa for PE/AgNP (1.05%), and PE/AgNP (3.10%), respectively, which represent increases of about 4% and 19%. In Fig. 2(b), RDF profiles reveal the PE density within 12 Å from the AgNP surface increases significantly and that within 12~15 Å slightly increases. For PE/AgNP (3.10%), the distance between the surfaces of two nearest AgNPs is about 27 Å along the tensile direction as shown in Fig. 1(e). Consequently, the mechanical properties of PE between AgNPs are enhanced, resulting in a significant improvement on the ultimate strength for PE/AgNP (3.10%) when compared to the improvement content of PE/AgNP (1.05%).

In order to examine the local structural rearrangements, atomic local shear strain ηi^Mises^ for an individual atom, a characterization developed by Shimizu *et al*.^59^, was used for its ability to monitor the formation of shear transition zones (STZ). Details of ηi^Mises^ are presented in reference^60^ and is therefore not introduced here. Larger ηi^Mises^ values indicate that an atom i is under local plastic and shear deformation; in contrast, a small ηi^Mises^ value indicates either that atom i is undergoing only a small amount of movement when considered relative to all its first neighbor atoms, or that atom i can be considered to be under local elastic deformation. Distributions for atomic local shear strain in this system were calculated, and are presented using the software OVITO. Structures at a strain value of 0 are considered to be references for subsequent calculations of atomic local shear strains under different tensile strains in all three structures.

**Figure 4:**
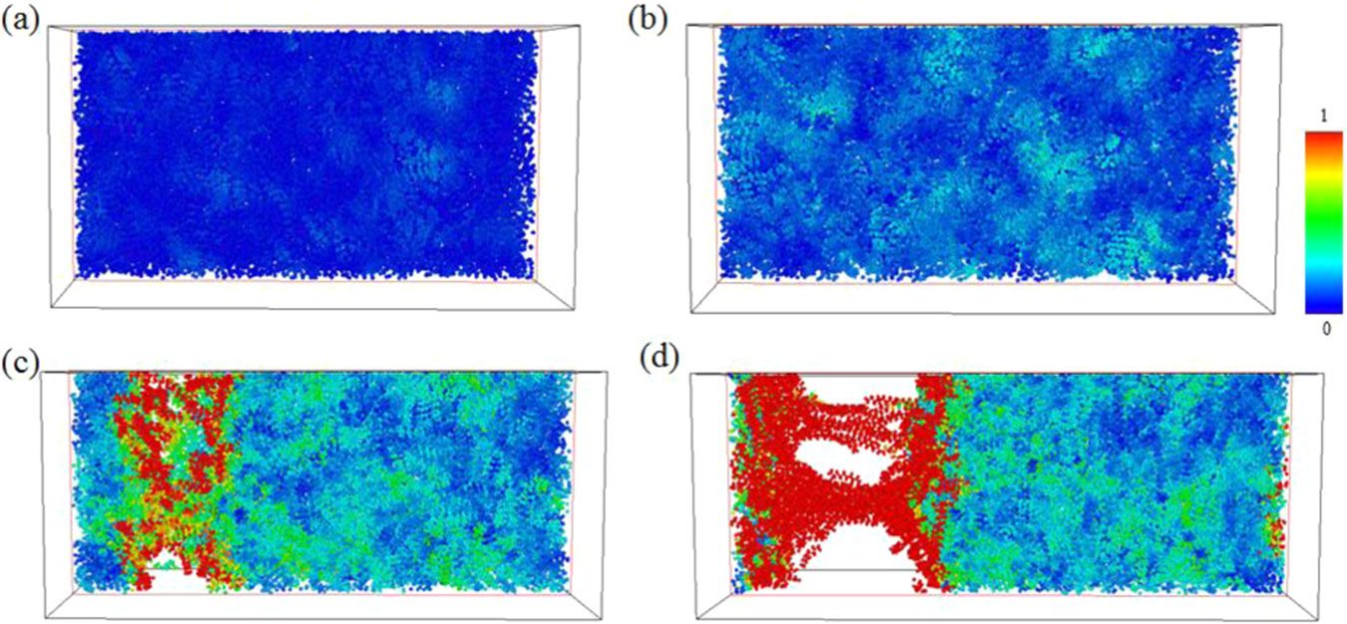
Snapshots of the uniaxial tensile deformation in pristine PE at strain = (**a**) 0.02, (**b**) 0.13 (ultimate stress), (**c**) 0.25, and (**d**) 0.4. Atoms are colored corresponding to their local shear strain values relative to the reference structure at strain 0.

Figure 4(a–d) shows atomic η_i_^Mises^ values for pure PE at strains of 0.02, 0.13 (at ultimate stress), 0.25, and 0.4. In the η_i_^Mises^ strain distribution = 0.02, (Fig. 4(a)) which is the upper bound to calculate Young’s modulus, the η_i_^Mises^ values of most atoms are near 0, which indicates that, at this particular strain value, the local elastic deformation is dominant. At strain value of ultimate stress, (Fig. 4(b)) some local regions containing atoms with larger η_i_^Mises^ values are distributed randomly throughout the pristine PE. After the strain exceeds the value corresponding to the ultimate stress, empty space, or voids, appear within the PE, and the atoms surrounding these voids are subject to quite serious local shear deformation, as has been illustrated in Fig. 4(c). Finally, at strain of 0.4, these voids exhibit continuous growth from those found at the strain of 0.25, leading to a fracture in the PE.

**Figure 5:**
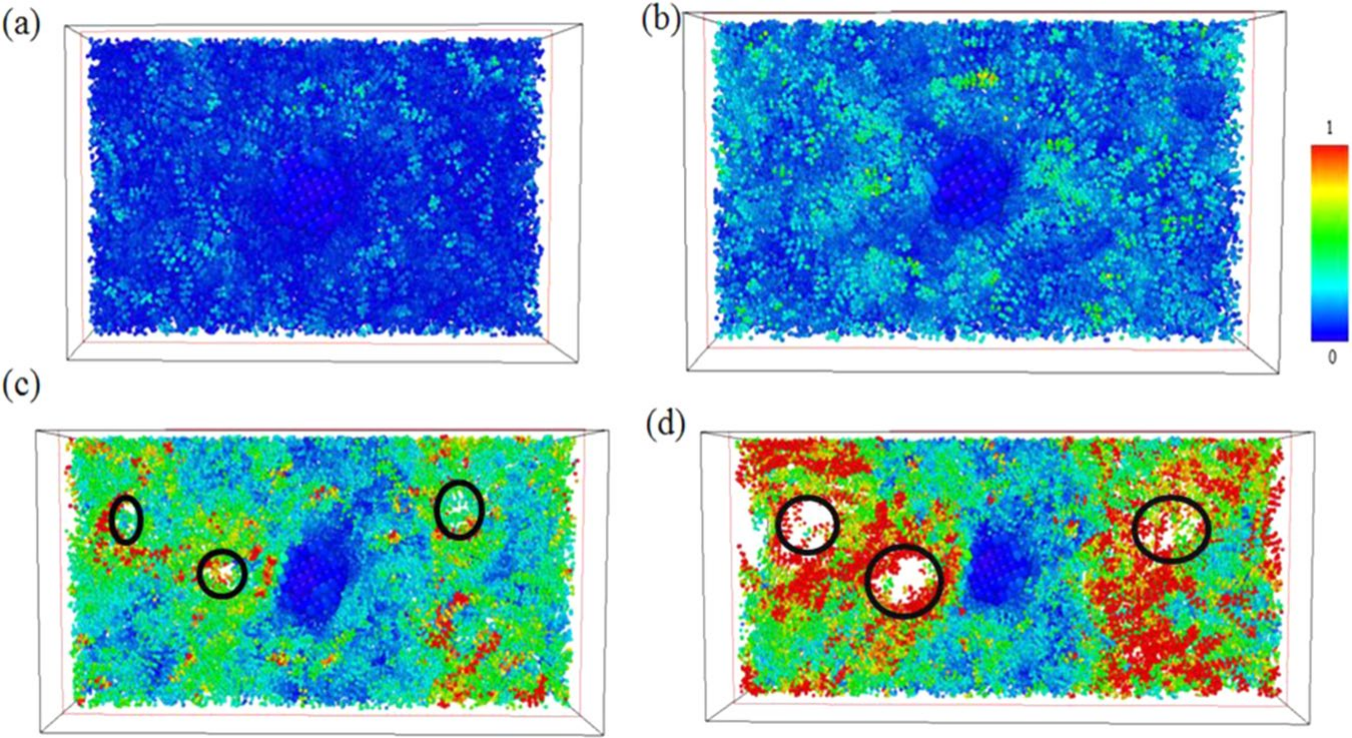
Snapshots of uniaxial tensile deformation in PE/AgNP (1.05%) at strain = (**a**) 0.02, (**b**) 0.13 (ultimate stress), (**c**) 0.25, and (**d**) 0.4. Atoms are colored corresponding to their local shear strain values relative to the reference structure at strain 0.

**Figure 6:**
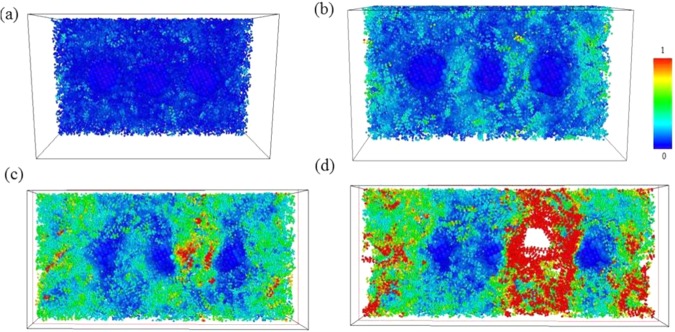
Snapshots of uniaxial tensile deformation in PE/AgNP (3.10%) for strain = (**a**) 0.02, (**b**) 0.13 (ultimate stress), (**c**) 0.25, and (**d**) 0.4. Atoms are colored corresponding to their local shear strain values relative to the reference structure at strain 0.

For PE/AgNP (1.05%) and PE/AgNP (3.10%), shown in Figs. 5 and 6, the distributions of η_i_^Mises^ values near the AgNPs are near zero during the tensile process, indicating that the PE atoms are under local elastic deformation, even though the local structures of these two PE/AgNP composites are seriously deformed and fracture when strain is larger than the ultimate stress point. As is evident in Fig. 2(b), the interactions between the AgNP and PE near the AgNP surface has led to a higher PE density around the AgNPs, which in turn also produces an increase in the local strength of PE surrounding the AgNPs. For PE/AgNP (1.05%), because the distance of influence of the AgNP surface on the PE density is about 12 Å, according to the RDF result, the voids appear at locations over 12 Å away from the AgNP surface, which have been circled in Fig. 5(c). These voids become larger with increasing strain, as shown in Fig. 5(d).

For PE/AgNP (3.10%), three AgNPs are well dispersed within the PE matrix, and most PE chains in the system are affected by the AgNPs. Consequently, the strength of this PE composite is higher than those of other cases, and this can be seen in Fig. 6 (c), where no void appears within the PE matrix at a strain of 0.25, unlike the voids shown in Fig. 4(c) and 5(c) for pristine PE and PE/AgNP (1.05%). At strain of 0.4, a void can be seen between the two AgNPs.

**Figure 7:**
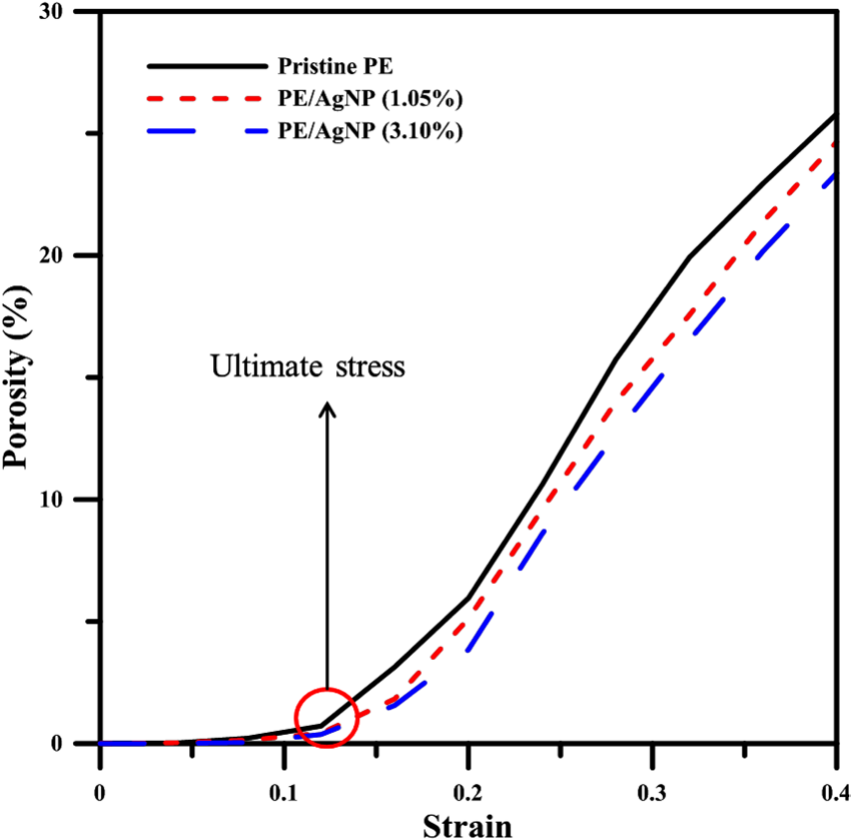
Profiles of the porosity percentage for pristine PE, PE/AgNP (1.05%), and PE/AgNP (3.10%) at different strains.

To better study variations in the porosity of each system during this tensile strain process, the porosity of the system is calculated as:^58^

3 $${\rm{Porosity}}\,=\,\frac{{\rm{total}}\,{\rm{volume}}\,-\,{\rm{solid}}\,{\rm{volume}}}{{\rm{total}}\,{\rm{volume}}} \% $$

where the total volume is the volume of the simulation system and the solid volume is the volume per one atom, as determined by OVITO with a probe size of 4 Å. Figure 7 shows the percentage of porosity for all three cases at different strain values. The porosity percentages at strain values smaller than 0.04 are near zero, which indicates that, at this strain range, the local damage is insignificant. When strain increases from 0.04 to that of the ultimate stress point, porosity percentage increases slightly with increasing tensile strain. For strains larger than those at the ultimate stress point, this percentage increases significantly with increasing strain. Over this strain range, the porosity of pristine PE at the same strain is higher than those of either PE/AgNP (1.05%) or PE/AgNP (3.10%), which exhibit a lower percentage of porosity at higher Ag wt%. A comparison of the fracture mechanism which is shown in Figs. 4–6 confirms that the size of the voids within the PE matrix become relatively smaller at a higher Ag wt%. This occurs due to the interaction between the PE and AgNP enhancing the local strength of PE. As a result of this, the porosity at a lower Ag wt% becomes relatively higher, resulting in a lower PE strength.

In order to investigate the increase in the excess local volume during this tensile strain process, the increase in free volume was calculated as per the definition of free volume increase percentage (FVIP), which is:

4 $${\rm{FVIP}}(\varepsilon )=\frac{{\sum }_{i=1}^{N}{V}_{i}(\varepsilon )-{\sum }_{i=1}^{N}{V}_{i}(0)}{{\sum }_{i=1}^{N}{V}_{i}(0)}$$

where ${V}_{i}(\varepsilon )$ is the volume of atom *i* at strain of $\varepsilon$, and ${V}_{i}(0)$ is the initial volume of the system at a strain of 0. In order to obtain the atom volume created by the reference atom with its first neighbour atoms, the following equation, as has been derived by Srolovitz, was used^61^:

5 $${V}_{i}=\frac{4\pi }{3}{a}_{i}^{3},{a}_{i}=\frac{\sum {r}_{ij}^{-1}}{2\sum {r}_{ij}^{-2}}$$

where a_*i*_ is the average radius of atom *i*, and *r*_*ij*_ is the distance between atom *i* and its neighbor atom *j*. Note that the first non-bonded neighbour atoms of a reference atom at strain 0 were used to calculate the atom’s volume during the tensile process.

**Figure 8:**
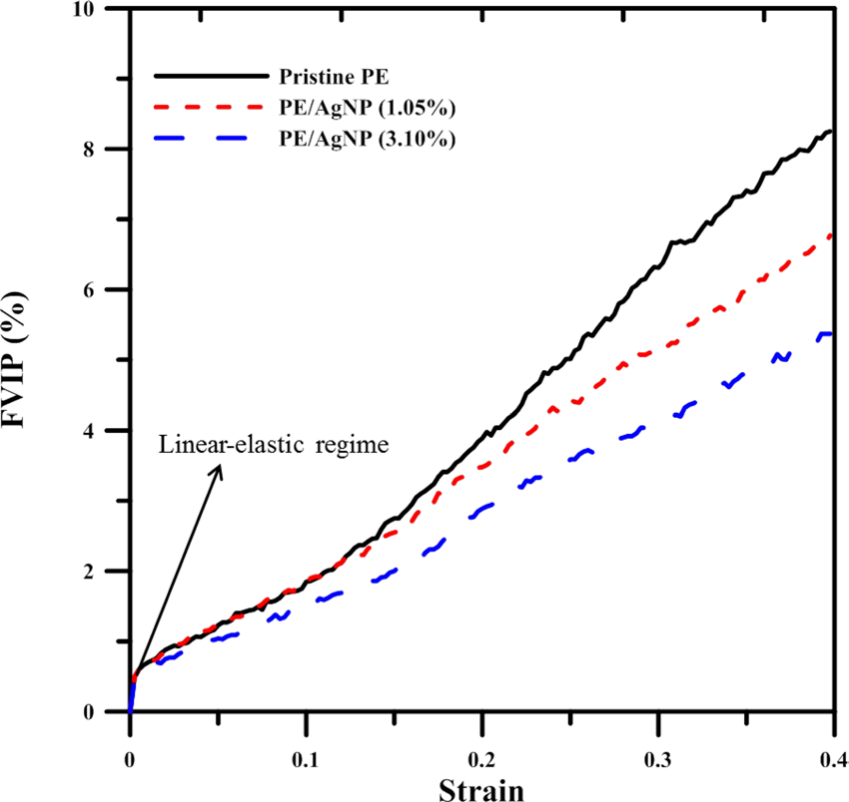
Profiles showing increase in percentage of free volume for pristine PE, PE/AgNP (1.05%), and PE/AgNP (3.10%) at different strains.

Figure 8 shows the resultant FVIP profiles at different strain values for the pristine PE, as well as those for the PE/AgNP (1.05%) and (3.10%). In all profiles, the FVIP exhibits a distinct linear jump from 0 to 0.05, which occurs when the strain increases from 0 to 0.02. Figures 4(a), 5(a) and 6(a) show that most atoms have small local shear strain values, which indicate that the volume of most atoms undergo elastic expansion at this strain range. In all cases, as the strain continuously increases from 0.02, the FVIP also increases. Further, at strains lower than that of ultimate strain, the FVIP profile for pristine PE in fact closely matches that of PE/AgNP (1.05%). When strain becomes higher than that at the point of ultimate stress, the FVIP for pristine PE demonstrates increases which are more significant than those found in either PE/AgNP composite. This, therefore, indicates that the void sizes in the pristine PE become more pronounced with an increase in strain, as is seen in Figs. 4(d), 5(d), and 6(d). The FVIP of PE/AgNP (3.10%) is in fact the lowest among these three cases at all strains, because the interaction between the AgNPs and PE chains improves the strength of the PE matrix considerably more than does that of the PE/AgNP (1.05%) matrix.

In order to explore the local structural variations present around different types of atoms, it is necessary to determine the variations in the average distance of all non-bonded first neighbor atoms from a reference atom, for each of the different atom types. The definition of the first neighbor distance variation of atom _*i*_ is given as:

6 $${R}_{i}(\varepsilon )=\frac{{\sum }_{j}{r}_{ij}(\varepsilon )-{\sum }_{j}{r}_{ij}(0)}{{\sum }_{j}{r}_{ij}(0)}$$

where $\,\sum _{j}{r}_{ij}(\varepsilon )$ is the summation of the distances between the reference atom *i* and all its non-bonded first neighbor atoms *j* at strain of $\varepsilon$, and $\sum _{j}{r}_{ij}(0)$ is the sum of the distances at strain of 0. It should be noted the first neighbor distances of different pairs used in Eq. (6) are determined from the distances at the minimums between the first two RDF peaks. The values of the first neighbor distance variations were also averaged for the last 5 ps of 20 ps relaxation process before applying the next tensile increment.

**Figure 9:**
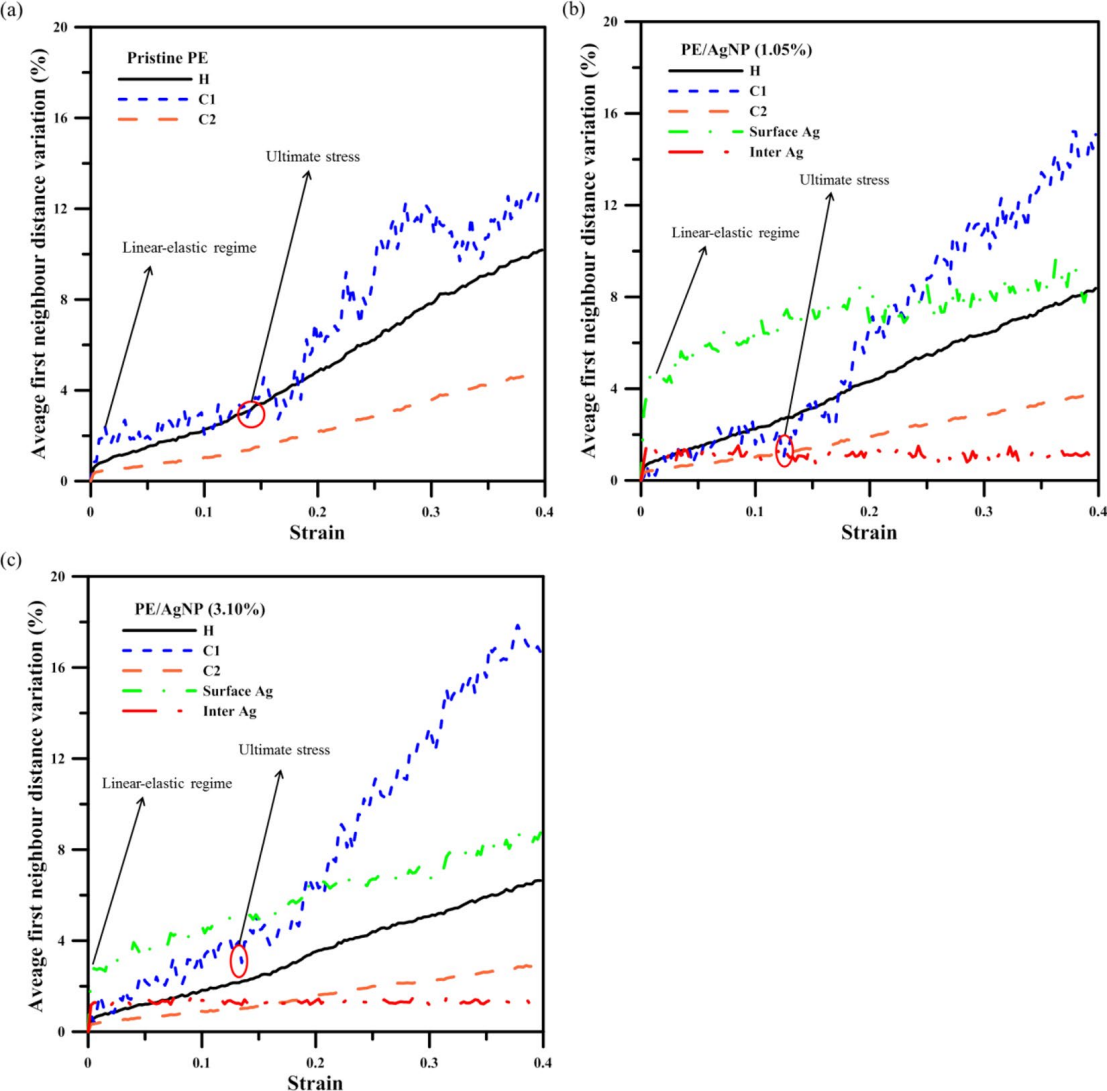
Profiles of variation in average first neighbor distance in (**a**) pristine PE, (**b**) PE/AgNP (1.05%), and (**c**) PE/AgNP (3.10%) at different strains.

For convenience in presenting our calculation results, the symbols H, C1, and C2 are used to indicate the average distances of hydrogen atoms, of carbon atoms located between two PE terminal carbon atoms, and of the terminal carbon atoms of the PE chains, respectively. For the AgNP, the average distances were also calculated for the surface Ag atoms with the Ag coordination number lower than 12 and for the interior Ag atoms having the Ag coordination number of 12 that do not directly contact PE atoms. For the interior Ag atoms, the average first neighbor distance variation increases from 0 to about 1% at the strain of 0.02, and then fluctuates around a constant value of about 1% during the tensile process, indicating the shape of AgNP basically remains unchanged for the tensile load. For the surface Ag atoms, the profiles indicate that the variation in average first neighbor distance is most pronounced at a strain = 0.02, as is evident in Fig. 9(b,c). This confirms that the local atomic volume expansions for the surface Ag atoms are more significant in the elastic region. At strain values higher than 0.02, the profiles display a relatively slight increase with increasing strain when compared with those of H, C1, and C2.

The effect of temperature on mechanical properties of PE and PE/AgNP composite was also investigated using MD simulation. Here, only the simulation results for pristine PE and PE/AgNP (3.10%) are discussed. The NPT ensemble was applied to the temperature elevation process from 100 K to 800 K at 1 atm. 10 ps was adopted to make sure the system reaches the thermal equilibrium before applying the next temperature increment of 25 K. Consequently, the heating rate is 25 K/10 ps, below which the MD simulation results are almost indistinguishable. In order to obtain melting temperatures (T_m_) for pristine PE and PE/AgNP (3.10%), their system volumes were sampled at different temperatures in the range 100 to 800 K.

**Figure 10:**
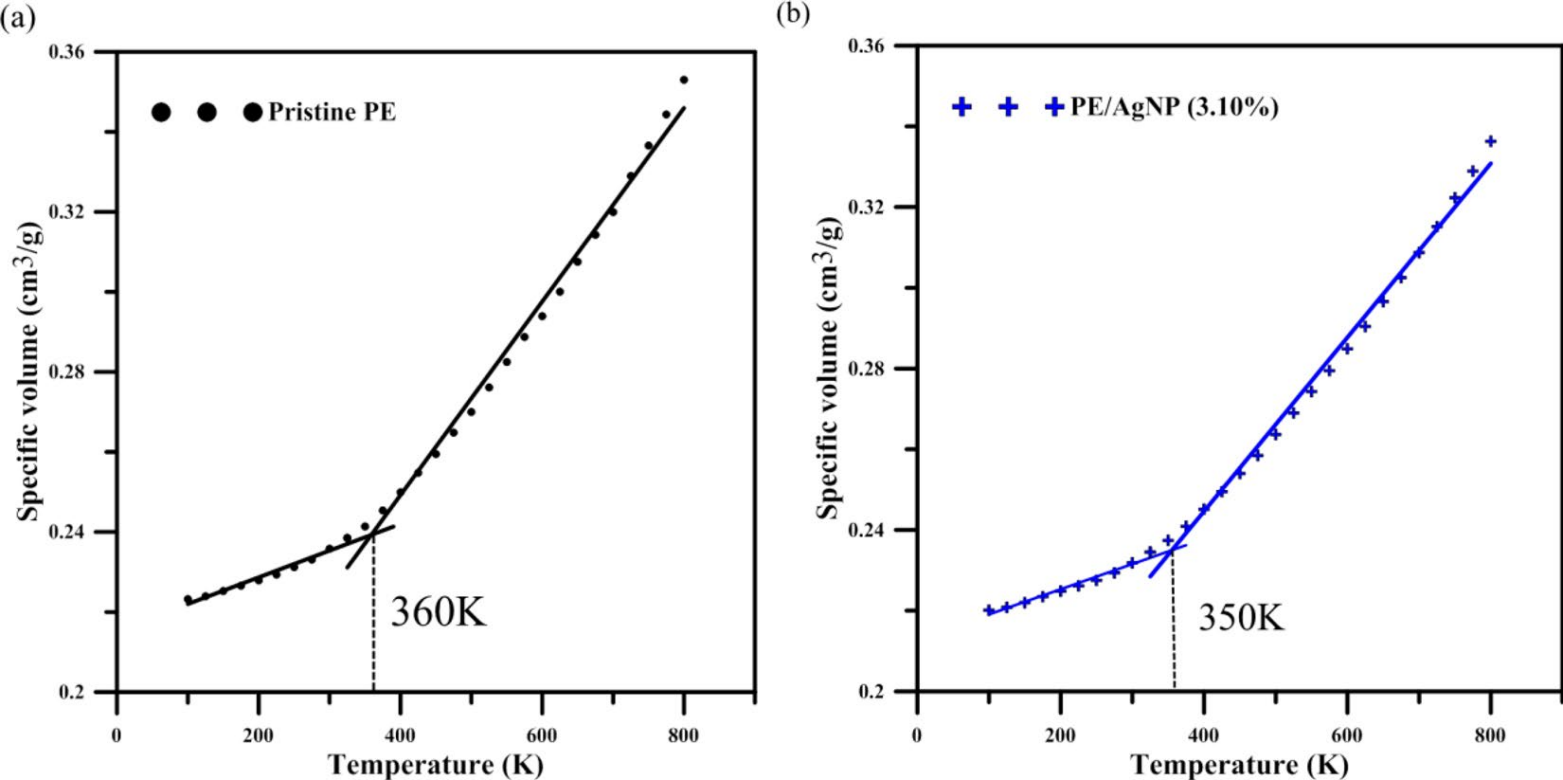
Specific volume with temperature during the temperature elevation process for (**a**) pristine PE and (**b**) PE/AgNP (3.10%).

Figure 10 shows specific volume profiles with temperature. The profiles exhibit a linear increase from 100 K to the specific temperatures, and then increase with a sharper slope at temperatures exceeding these specific temperatures. Melting temperature can be determined by the intersection of two lines plotted from the initial (100–300 K) and final ranges (370–800 K), and is indicated with a dashed line in Fig. 10. The predicted melting temperature of PE is about 360 K, close to the experimental value range of 380~409 K^62^. For the AgNP composite, the predicted melting point is lower than that of pristine PE by 10 K.

**Figure 11:**
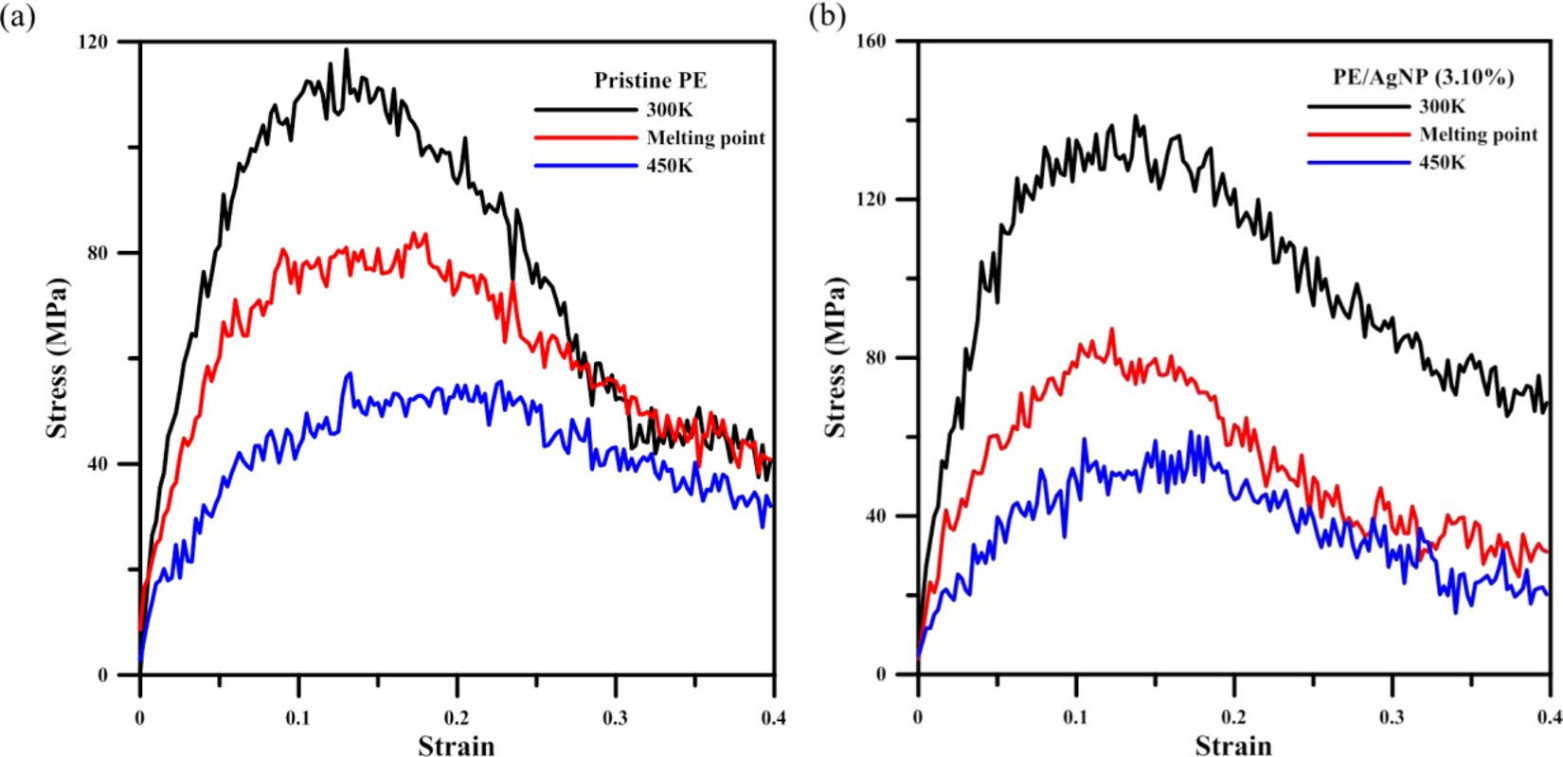
Stress-strain curves for (**a**) PE and (**b**) PE/AgNP (3.10%) at 300K, 350K, and 450K.

To better understand the effect of temperature on the mechanical properties, tensile simulations were carried out both at their melting points, as well as at temperatures higher than these melting points. Figure 11 shows the stress-strain curves for the pristine PE and PE/AgNP (3.10%) under tensile loading at temperatures of 300 K, 350 K, and 450 K. The figure demonstrates that ultimate tensile strength and Young’s modulus of PE at 300 K are higher than those at 350 K and 450 K for both cases, indicating the temperature in fact has a strong effect on the mechanical properties of both PE and PE/AgNP composites, with the mechanical properties undergoing a significant reduction at higher temperatures.

Details of the mechanical properties of pristine PE and PE/AgNP (3.10%) derived from the stress-strain profiles of Fig. 11(a,b) have been listed in Table 5. For pristine PE at temperatures of 300 K, 350 K, and 450 K, the Young’s moduli are about 1495.16 MPa, 980.75 MPa, and 520.65 MPa, respectively. At 350 K and 450 K, this represents reductions of about 34% and 65% from that of pristine PE at 300 K. Table 5 also shows that the tensile strength can be reduced from 118.55 MPa to 83.74 MPa at 350 K and 57.18 MPa at 450 K, decreases of about 29% and 52% from that of pristine PE at 300 K.

**Table 5:** Young’s modulus and ultimate stress of pristine PE and PE/AgNP (3.10%) at different temperatures.

	PE			PE/AgNP (3.10%)		
Temperature (K)	300	350	450	300	350	450
Young’s modulus (MPa)	1495.16	980.75	520.65	1830.76	1035.55	537.60
Ultimate stress (MPa)	118.55	83.74	57.18	141.09	87.32	61.36

Changes in PE/AgNP (3.10%) with temperature follow a similar pattern. At 350 K and 450 K, the Young’s moduli are about 1035.55 MPa and 537.60 MPa, representing a decrease of about 43% and 71% from PE/AgNP (3.10%) at 300 K. Additionally, it can be seen that the ultimate tensile strength is reduced from 141.09 MPa to 87.32 MPa and 61.36 MPa at 350 K and 450 K, respectively, a decrease of about 38% and 57%. Comparing the Young’s modulus and ultimate strength of pristine PE to those of PE/AgNP (3.10%), it is clear that the Young’s moduli and ultimate strengths of pristine PE and PE/AgNP (3.10%) are very close once the system temperature reaches the melting point of PE, even though these two mechanical properties of PE/AgNP (3.10%) are relatively higher than those of pristine PE at 300 K.

**Figure 12:**
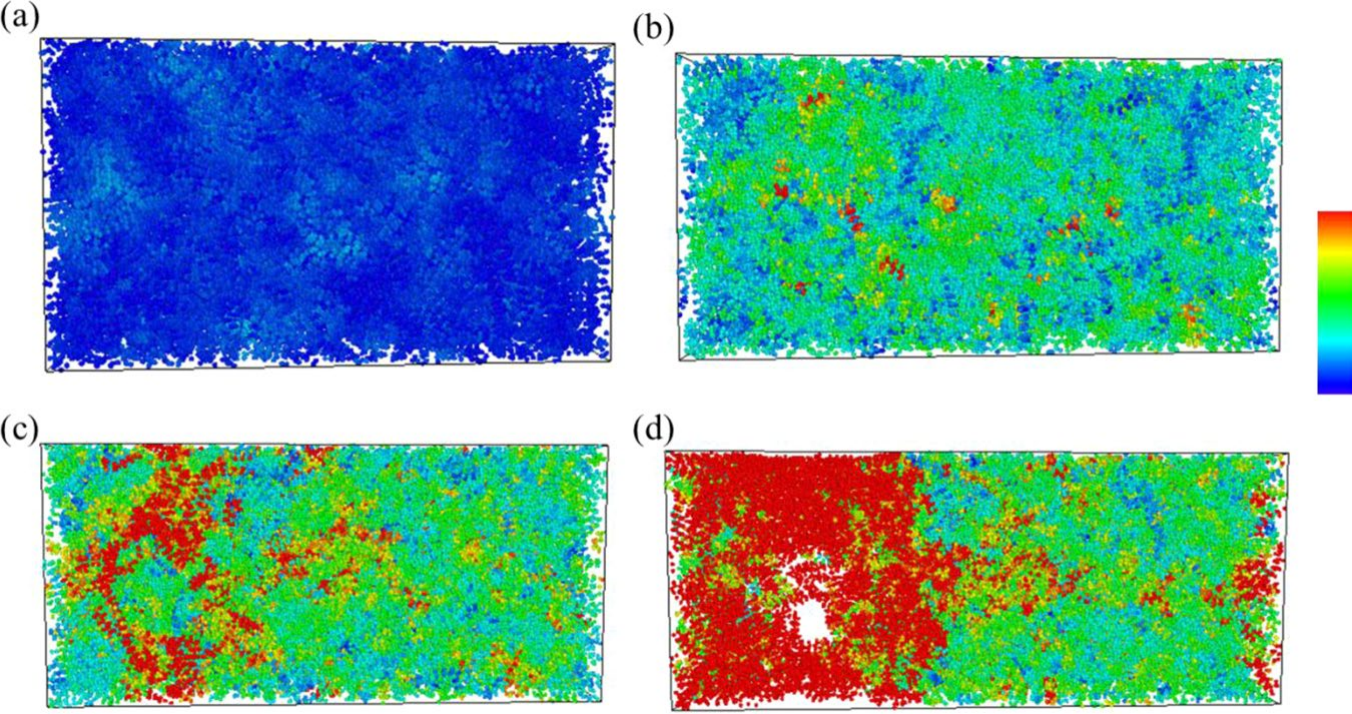
Uniaxial tensile deformation snapshots of pristine PE at strains of (**a**) 0.02, **(b**) 0.1225 (ultimate stress), (**c**) 0.25, and (**d**) 0.4 at 350K. Atoms are colored with their corresponding local shear strain values relative to the reference structure at strain 0.

Figure 12 shows snapshots of pristine PE with their atomic η_i_^Mises^ values at strain values of 0.02, 0.1225 (ultimate stress), 0.25, and 0.4 at 350 K. Compared to the corresponding strains for pristine PE at 300 K shown in Figs. 4(a–d), 12(d) exhibits a relatively smaller void than that in Fig. 4(d). This indicates that the pristine PE becomes more ductile as temperature draws close to its melting point.

**Figure 13:**
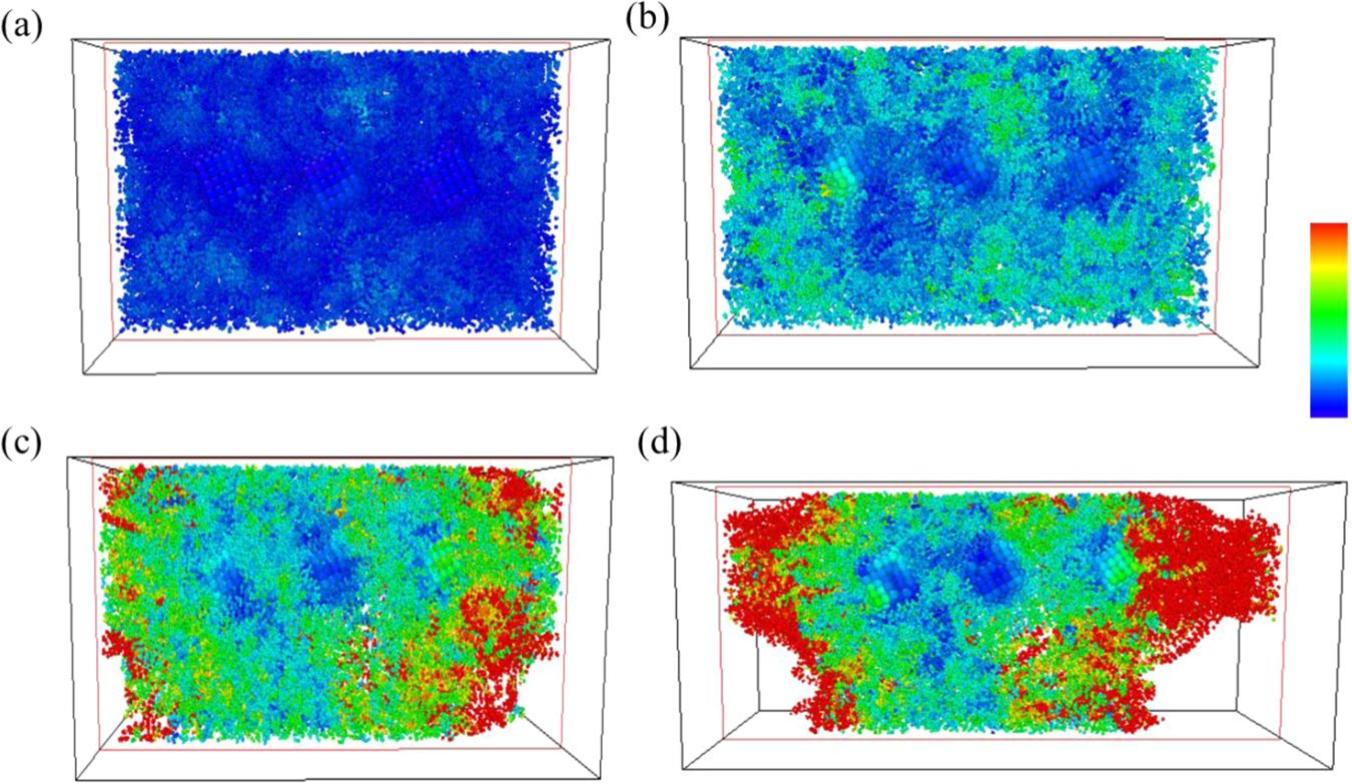
Snapshots of uniaxial tensile deformation in PE/AgNP (3.10%) at strains of (**a**) 0.02, (**b**) 0.1225 (ultimate stress), (**c**) 0.25, and (**d**) 0.4 at the melting point of 350 K. Atoms are colored with their corresponding local shear strain values relative to the referenced structure at strain 0.

For PE/AgNP (3.10%) at 350 K, Fig. 13 indicates the AgNPs possess higher mobility within the PE matrix as compared to those shown in Fig. 6(a–d). These AgNPs move closer together with an increase in strain, and a void appears in the region only containing PE, as in Fig. 13(c). Although the strength of PE in the vicinity of the AgNPs was enhanced, the tensile behavior at the PE region was very similar to that of pristine PE. Consequently, the mechanical properties of PE/AgNP (3.10%) are very similar to those of pristine PE when the temperature exceeds their melting points.

## Conclusions

The mechanical behaviors of PE and PE/AgNP composites at different weight fractions of Ag were studied using molecular dynamics tensile simulations. As can be seen from the RDF profile at strain of 0, the interaction strength between the AgNP surface and the PE chain is strong, and the interaction distance is long, resulting in a higher local PE density around 12 Å from the AgNP surface. At 300 K, the tensile simulation shows that the Young’s modulus and strength of the PE matrix were considerably enhanced with increasing AgNP fraction. The increases in Young’s moduli of PE/AgNP (1.05%) and PE/AgNP (3.10%) are about 11% and 18% larger than that of pristine PE, and the tensile strength can be enhanced from 118.55 MPa for pristine PE to 123.56 MPa and 141.09 MPa for PE/AgNP (1.05%) and PE/AgNP (3.10%), respectively.

The ηi^Mises^ evolution under different strains indicates that the ηi^Mises^ values for the PE atoms near the AgNP surface remain relatively lower during tensile strain, which indicates that the PE atoms located in this region are still in a state of local elastic deformation, even though the PE/AgNP (1.05%) and PE/AgNP (3.10%) become seriously deformed and fracture at strain larger than that at ultimate stress. Investigating variation in average first neighbor distance shows that the variation in surface Ag atoms of AgNP is more significant in the elastic region. As the strain increases from 0.02, the profiles display a relatively slight increase for surface Ag atoms when compared to those of H, C1, and C2 atoms. Investigation of the ηi^Mises^ distributions and the average first neighbor distance variation profiles at different strains indicates that the improvement in the mechanical properties of PE/AgNP (1.05%) and PE/AgNP (3.10%) can be attributed to the enhancement of local PE structures around the AgNPs.

To examine the effect of temperature on the mechanical properties of pristine PE and PE/AgNP (3.10%), tensile simulations were carried out at 350 K and 450 K, temperatures close to the predicted melting point of pristine PE, that of about 360 K. The temperature elevation process was operated from 300 K to 800 K. The simulation results show that at 350 K and 450 K, PE materials become more ductile than at 300 K, because voids begin to appear at higher tensile strains. As temperature increases, the Young’s modulus and ultimate strength of PE exhibit a significant decrease. For PE/AgNP (3.10%) at 350 K and 450 K, the AgNPs within the PE matrix exhibit higher mobility than those at 300 K, and the AgNPs became closer as strain increases. Therefore, the fracture occurs in the PE part of PE/AgNP (3.10%), resulting in a similar Young’s modulus and ultimate strengths in both pristine PE and PE/AgNP (3.10%) at 350 K and 450 K.

Based on the current MD results for PE/AgNP composites, the mechanical property improvement of pristine PE by adding AgNPs as observed in the experiments^41–43^ can be reflected by the slopes of stress-strain profiles. The reliability on the mechanical property prediction by the MD simulation can also be seen for other polymer/nano-filler composites in Refs. ^30–35^,^44–46^. According to the stress variation at different strains, the distributions of atomic local shear strain ηiMises at elastic, plastic, ultimate stress regions clearly show the local structural evolution during the tensile process within the PE matrix and AgNP as well as the interface of PE/AgNP. This per-atom parameter used originally for bulk metallic glass (BMG) can provide a clear picture of local deformation around each atom and has not been used in previous related polymer/nano-filler studies. We believe the current molecular simulation process can not only understand the material property improvement mechanism of polymer/nano-filler composites, but also provide a reliable numerical method to develop new polymer/nano-filler composites.
